# Expansion microscopy with carboxylic trifunctional linkers

**DOI:** 10.3724/abbs.2022113

**Published:** 2022-08-22

**Authors:** Xuecheng Chen, Yaqian Li, Jiabin Wang, Jielin Sun, Daniel M. Czajkowsky, Zhifeng Shao

**Affiliations:** 1 Shanghai Center for Systems Biomedicine Shanghai Jiao Tong University Shanghai 200240 China; 2 State Key Laboratory for Oncogenes and Bio-ID Center School of Biomedical Engineering Shanghai Jiao Tong University Shanghai 200240 China

Super-resolution optical microscopy has fundam entally transformed structural biology by enabling characterization of biological complexes with up to tenfold greater resolution than conventional microscopy [1]. Among the number of different approaches with super-resolution capability, there is perhaps none more generally accessible to most research labs than expansion microscopy (ExM), where the enhanced resolution is owing to the physical separation of fluorescent signals (typically at least 4-fold) and imaging is performed using conventional optical or confocal microscopy [2]. Combining ExM with other super-resolution methods, such as structured illumination microscopy (SIM), has been shown to further improve resolution down to 20 to 30 nm [3]. However, it has also become apparent that two steps in the ExM procedure (namely, protein digestion and the polymerization of the acrylamide gel) lead to substantial fluorescence loss (>50%), which thus fundamentally reduces the resolving capacity of ExM [2]. Recently, a method was developed (Label-retention ExM) that overcomes this major obstacle [4]. This approach employs trifunctional anchors that are impervious to proteolysis as well as the addition of fluorophores post-expansion, which lead to a nearly 6-fold increase in fluorescence intensity from conventional ExM. Still, while this development is indeed significant, the synthesis of the trifunctional anchors requires considerable expertise in organic chemistry as well as access to techniques (such as TLC, NMR and mass spectroscopy) for the analysis of the products and intermediates during synthesis, which essentially puts this method out of the reach of most biological labs.

Here we present a simple method, called carboxylic trifunctional linkers-ExM (CT-ExM), in which trifunctional linkers are produced by a rapid (1 day) process using commercially available materials and routine reaction schemes. As such, we believe we now make this strategy of improving fluorescence more widely accessible to many biological labs, and thus ultimately its application in structural biology.

The basic synthesis of our trifunctional linkers (TLs) is depicted in Figure 1. In short, unconjugated antibodies are modified with a DBCO-NHS ester and then purified using a simple spin desalting column. Separately, commercial PEG (polyethylene glycol) linkers and hapten-NHS esters are simply mixed and stirred overnight to obtain the hapten-TLs. The hapten-TLs and DBCO-antibodies are then mixed to undergo a copper-free cycloaddition reaction, followed by purification with the desalting column to finally obtain the hapten-TL-modified antibodies ( Figure 1A,B). To anchor the antibodies to the hydrogel, the carboxylic TLs undergo an EDC activation reaction with 2-aminoethyl methacrylate (AEMA) (instead of the commonly used acryloyl NHS esters) ( Figure 1C,D). Thus, all of the procedures in our approach take advantage of already well-established methods commonly used in many labs.

**Figure 1:**
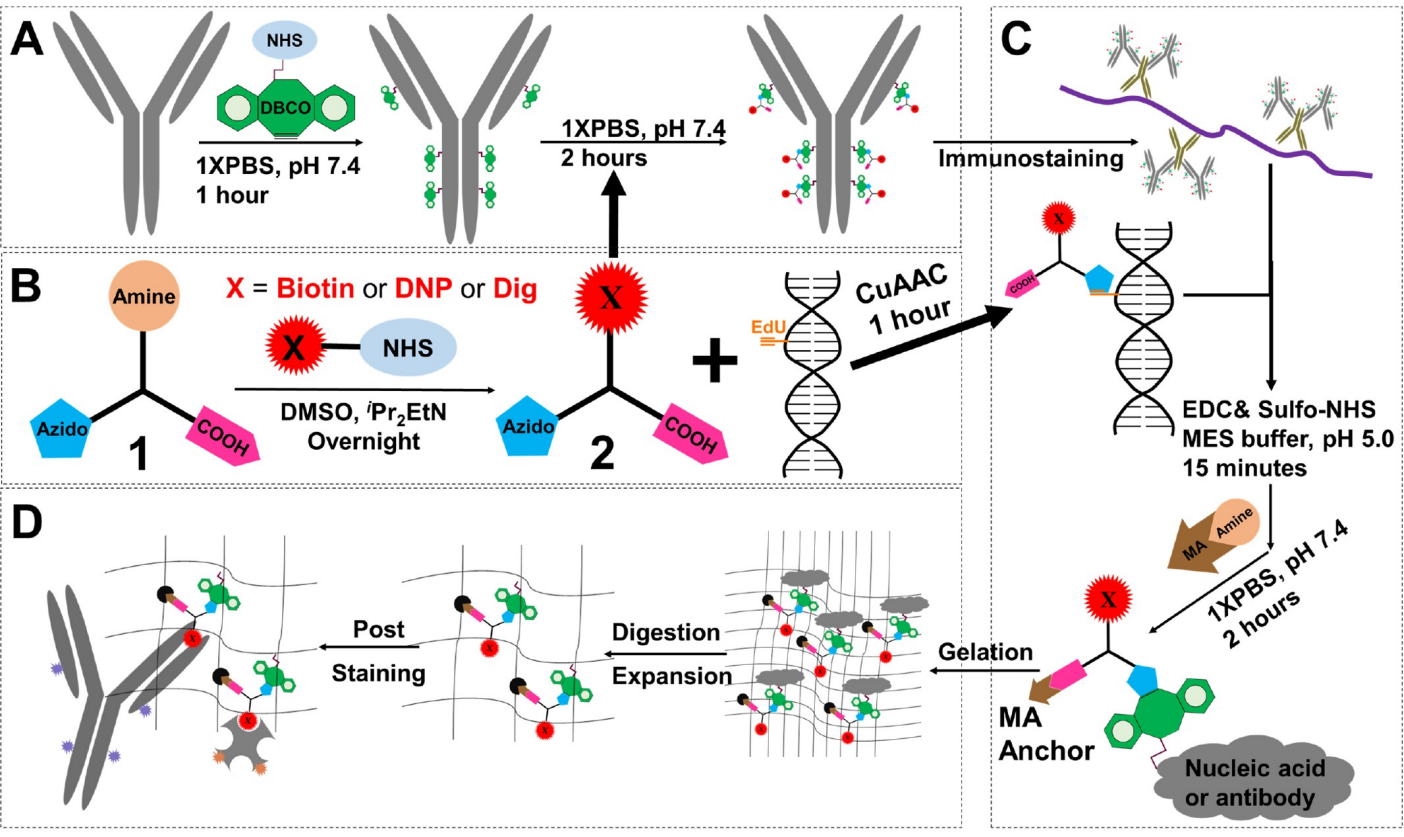
Figure 1 Schematic diagram of CT-ExM with tri-functional linkers (A) The antibody is modified with DBCO-NHS ester (green). (B) Synthesis of carboxylic trifunctional linkers (azido (blue), carboxylic acid (pink) and hapten X (red)). (C) Immunostaining or the click reaction labels the sample, and EDC activation of carboxylic acid, followed by addition of a methylacrylate group enables subsequent attachment to the gel. (D) Expansion Microscopy and post-expansion labeling of haptens with fluorescently-labeled antibodies or streptavidin.

To demonstrate the effectiveness of our approach, we first studied microtubule filaments in COS-7 cells ( Figure 2A), immunostaining with primary antibodies (T6199, 1:1000; Sigma-Aldrich, St Louis, USA) and dinitrophenol (DNP)-TL-labeled secondary antibodies (modified from M8642, 1:200; Sigma-Aldrich). Following ~5-fold expansion ( Supplementary Figure S1A) with negligible distortion ( Supplementary Figure S1B), the sample was incubated with anti-DNP primary antibodies (04-8300, 1:200; ThermoFisher, Waltham, USA) and fluorescently-conjugated secondary antibodies (A48272, 1:500; ThermoFisher) and then imaged with 3D-SIM [5] ( Supplementary Figure S1D,E). The images showed the common cellular distribution of microtubule filaments, without any structural distortion ( Supplementary Figure S1B). Examination of the cross-sectional intensity along individual filaments revealed a bimodal distribution ( Figure 2B,C and Supplementary Figure S1C and S2A) that is commonly observed in super-resolution microscopy studies of these filaments, which is a consequence of the antibodies binding to the hollow microtubule filaments [6]. The peak-to-peak distances measured from 20 filaments is 253±40 nm ( Figure 2D), which thus corresponds to an unexpanded distance of ~50 nm, in excellent agreement with previous studies [6]. We confirmed this measurement and further demonstrated the versatility of our method, by using digoxiegenin (DIG)-TL-labeled secondary antibodies (modified from M8642, 1:200; Sigma-Aldrich), yielding a similar peak-to-peak distance ( Supplementary Figure S2B). To further verify the ubiquity of our labeling scheme, we also generated biotin-TL-labeled nanobodies (which are ~10-fold smaller than IgG ( Supplementary Figure S2D) and labeled post-expansion with fluorescent streptavidin (S32355, 1:100; ThermoFisher). Images of a similar quality were obtained ( Supplementary Figure S2C), with a peak-to-peak distance from single filaments after expansion of 189±34 nm. This corresponds to an unexpanded distance of ~38 nm, in excellent agreement with earlier work, where the shorter distance is owing to the smaller size of the nanobodies compared to the antibodies [6]. Thus, overall, these results demonstrate the high efficiency and reproducibility of our method with many different hapten-TLs ( Supplementary Figure S3).

**Figure 2:**
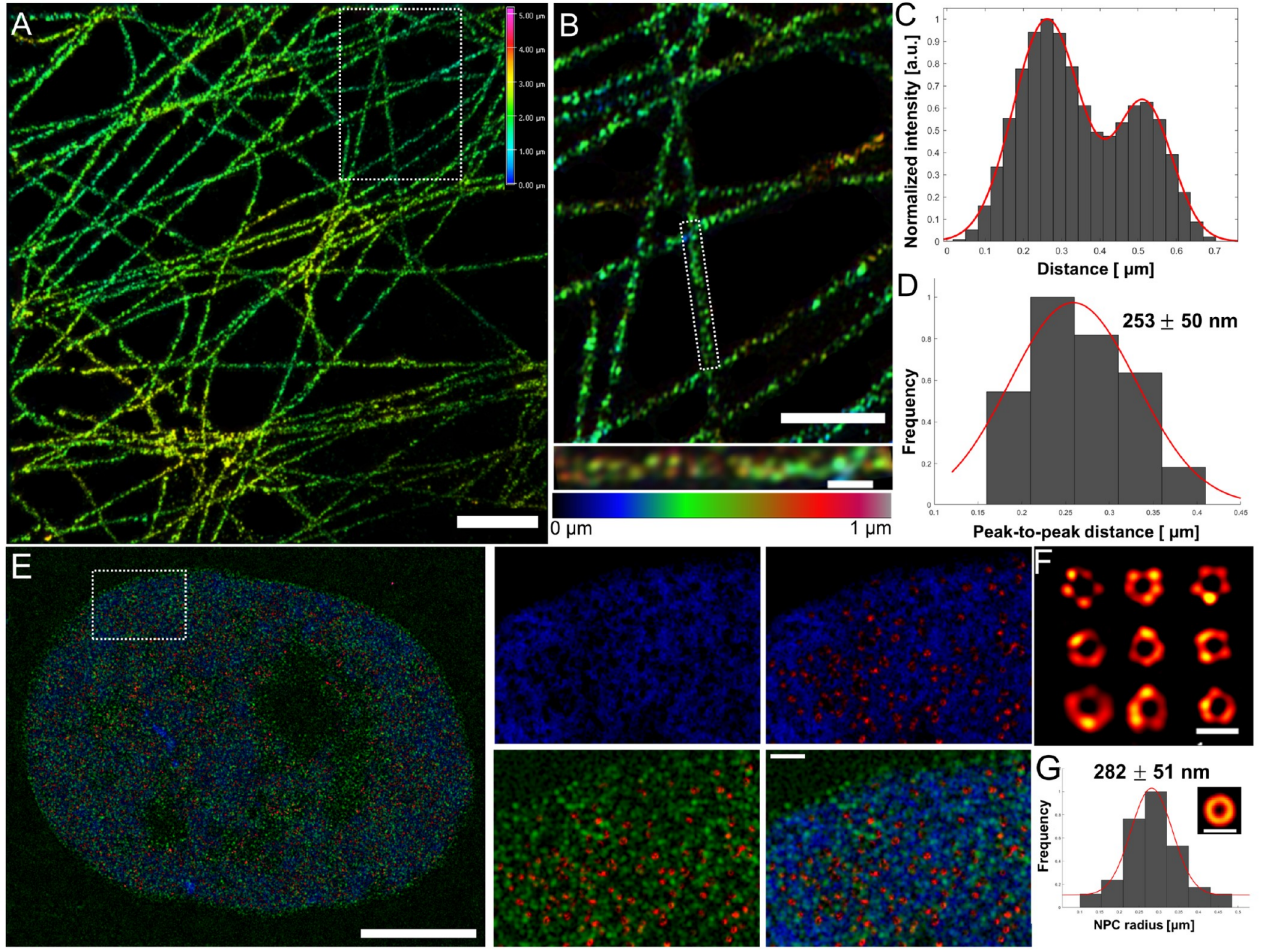
Figure 2 Application of CT-ExM to resolve cellular structures at sub-diffraction resolution All images were obtained with 3D-SIM. (A) Microtubules within COS-7 cells were labeled with a DNP-TL modified secondary antibody, then post-expansion stained with an anti-DNP antibody followed by Alexa 647-conjugated secondary antibody. Scale bar, 10 μm. (B) A magnified view of the boxed region in (A). The bottom panel is a magnified view of the single filament that is boxed in the top panel. Scale bar, top panel 5 μm, bottom panel 1 μm. (C) Signal intensity across the cross-section along the length of the filament shown in the bottom panel of (B). (D) Histogram of peak-to-peak distances together with a Gaussian-fitted curve (red) from multiple filaments ( n=20). (E) Multi-color labeling of EdU incorporated DNA (blue), NPCs (red) and nuclear lamina (green). The four panels on the right show the signals of the boxed region in the full composite panel on the left. Scale bar: left 25 μm, right 2.5 μm. (F) Collection of isolated individual Nup133 rings. Scale bar: 0.5 μm. (G) The radii of the individual Nup133 rings. The mean value 282±51 nm (mean±SD) corresponds to an unexpanded radius of 56 nm. The inset shows the averaged ring. Scale bar: 0.5 μm.

We finally demonstrated the versatility of our approach by simultaneously imaging DNA, nuclear pore complexes (NPC), and the nuclear lamina ( Figure 2E) in U2OS cells. The DNA was labeled by incorporating EdU (5-ethynyl-2′-deoxyuridine) into genomic DNA, followed by a click reaction with Biotin-TL, using a similar procedure as previously described [7]. The NPC protein, Nup133, and lamina protein, lamin B1, were labeled with DNP-TL- and DIG-TL-modified secondary antibodies (modified from M8642 and R2004, 1:200; Sigma-Aldrich) respectively. As shown in Figure 2E, the composite image reveals many intriguing correlative features between these components at a resolution of ~24 nm (see supplementary methods). In particular, the high-density of DNA near the nuclear envelope likely corresponds to heterochromatin while the low-density of DNA within the nuclear interior appear to form fibers of linked nanodomains ( Supplementary Figure S3B), in agreement with our previous work [7]. Further, at the apical cross-section of the nucleus, the DNA image exhibits many holes that are approximately the same size ( Figure 2E and Supplementary Figure S3A). Similar images in previous work were suggested to be owing to the presence of NPCs within the holes [8]. We now directly confirm this speculation with these images ( Figure 2E). In addition, we further find that holes within the lamina co-localize with the NPCs while the lamina itself overlaps with the DNA ( Figure 2E and Supplementary Figure S3B). Finally, we note that our images of the NPCs were of sufficient resolution to resolve individual subunits of the NPC complex ( Figure 2F and Supplementary Figure 4C). The density, as well as their size ( Figure 2G and Supplementary Figure S3D), are comparable to those obtained by single molecule localization microscopy [9] ( Supplementary Figure S4), which provides additional validation for the effectiveness of our CT-ExM method.

Thus, in summary, we have developed a simple, readily accessible approach to effectively attach labels onto the hydrogel for routine ExM. This idea builds on the approach introduced recently [4], but extends it significantly by markedly simplifying the synthesis of the TLs. Indeed, requiring reactions and components that are no more complicated than are typically used during, for example, the labeling of proteins with biotin or fluorophores, we believe our method can be easily performed in most biological labs with commonly available instrumentation. Whether labeling via antibodies, nanobodies, or via click chemistry, we anticipate that this approach will find wide use as a powerful method to interrogate biological structures at a resolution of tens of nanometers.

## Supplementary Data

Supplementary data is available at *Acta Biochimica et Biophysica Sinica* online.

## References

[1] Rust MJ, Bates M, Zhuang X (2006). Sub-diffraction-limit imaging by stochastic optical reconstruction microscopy (STORM). Nat Methods.

[2] Chen F, Tillberg PW, Boyden ES. Expansion microscopy.
*
Science
* 2015, 347: 543–538. https://doi.org/10.1126/science.1260088.

[3] Wang Y, Yu Z, Cahoon CK, Parmely T, Thomas N, Unruh JR, Slaughter BD (2018). Combined expansion microscopy with structured illumination microscopy for analyzing protein complexes. Nat Protoc.

[4] Shi X, Li Q, Dai Z, Tran AA, Feng S, Ramirez AD, Lin Z (2021). Label-retention expansion microscopy. J Cell Biol.

[5] Ball G, Demmerle J, Kaufmann R, Davis I, Dobbie IM, Schermelleh L (2015). SIMcheck: a toolbox for successful super-resolution structured illumination microscopy. Sci Rep.

[6] Zwettler FU, Reinhard S, Gambarotto D, Bell TD, Hamel V, Guichard P, et al. Molecular resolution imaging by post-labeling expansion single-molecule localization microscopy (Ex-SMLM).
*
Nat Commun
* 2020, 11: 1–11. https://doi.org/10.1101/2020.03.12.988923.

[7] Fang K, Chen X, Li X, Shen Y, Sun J, Czajkowsky DM, Shao Z (2018). Super-resolution imaging of individual human subchromosomal regions
*in situ* reveals nanoscopic building blocks of higher-order structure. ACS Nano.

[8] Schermelleh L, Carlton PM, Haase S, Shao L, Winoto L, Kner P, Burke B (2008). Subdiffraction multicolor imaging of the nuclear periphery with 3D structured illumination microscopy. Science.

[9] Szymborska A, de Marco A, Daigle N, Cordes VC, Briggs JAG, Ellenberg J (2013). Nuclear pore scaffold structure analyzed by super-resolution microscopy and particle averaging. Science.

